# Supplementary material for: LTA4H rs2660845 association with montelukast response in early and late-onset asthma
Source: PLoS One. 2021 Sep 22;16(9):e0257396. doi: 10.1371/journal.pone.0257396 (PMC8457475; doi:10.1371/journal.pone.0257396)
Supplement: S1 Table — GoSHARE (a) is the late-onset population (>18 years-old); GoSHARE (b) is the early-onset population (< = 18 years-old). (DOCX) [file pone.0257396.s001.docx]

**S1 Table. Power of the sample size for combined cohorts to detect increases in OR for asthma exacerbation.**

| Asthma onset | Ethnicity | Cohort | OR=1.2 | OR=1.5 | OR=3 |
| --- | --- | --- | --- | --- | --- |
| Late | Europeans | UKBiobank, GoSHARE (a) | 82% | 99% | 100% |
| Early | Europeans | GoSHARE (b), BREATHE, Tayside RCT, PAGES | 31% | 86% | 100% |
| Early | Europeans, African Americans, Hispanics/Latinos | GoSHARE (b), BREATHE, Tayside RCT, PAGES, SAGE, GALA II | 64% | 97% | 100% |

GoSHARE (a) is the late-onset population (>18 years-old)

GoSHARE (b) is the early-onset population (<= 18 years-old)
