# Supplementary material for: LTA4H rs2660845 association with montelukast response in early and late-onset asthma
Source: PLoS One. 2021 Sep 22;16(9):e0257396. doi: 10.1371/journal.pone.0257396 (PMC8457475; doi:10.1371/journal.pone.0257396)
Supplement: S2 Table — Step 1: inhaled short-acting β2-agonists (SABA) on demand; Step 2: regular inhaled steroids (ICS) plus SABA on demand; Step 3: regular inhaled long-acting β2-agonists (LABA) (salmeterol or formoterol) plus ICS with SABA on demand; Step 4: oral montelukast with SABA on demand (plus ICS plus/or regular LABA). (DOCX) [file pone.0257396.s002.docx]

**S2 Table. Selection of asthmatic patients in GoSHARE by treatment steps.**

| Steps | Medication | Number of GoSHARE patients (n) |
| --- | --- | --- |
| 1 | SABA as needed | 10,218 |
| 2 | SABA as needed +ICS | 5,388 |
| 3 | SABA as needed + ICS + LABA | 4,551 |
| 4 | SABA as needed + LTRA (+ICS +LABA) | 1,070 |

Step 1: inhaled short-acting β2-agonists (SABA) on demand;

Step 2: regular inhaled steroids (ICS) plus SABA on demand;

Step 3: regular inhaled long-acting β2-agonists (LABA) (salmeterol or formoterol) plus ICS with SABA on demand;

Step 4: oral montelukast with SABA on demand (plus ICS plus/or regular LABA)
