# Supplementary material for: LTA4H rs2660845 association with montelukast response in early and late-onset asthma
Source: PLoS One. 2021 Sep 22;16(9):e0257396. doi: 10.1371/journal.pone.0257396 (PMC8457475; doi:10.1371/journal.pone.0257396)
Supplement: S3 Table — For GALA II and SAGE, betas 95%(CI) are reported for quantitative variables; * Traits with a P <0.05 were used as covariates in the logistic regression. (DOCX) [file pone.0257396.s003.docx]

| S3 Table. Covariates association with asthma exacerbation binary trait.  *Traits with a P <0.05 were used as covariates in the logistic regression.  For GALA II and SAGE, betas 95%(CI) are reported for quantitative variables | | | | | | | | | |
| --- | --- | --- | --- | --- | --- | --- | --- | --- | --- |
|  |  | Odds ratio (95%CI) for exacerbation; P-value | | | | | Betas (95%CI) for exacerbation; P-value | | |
|  | UKBiobank  N=1,561 | GoSHARE (a)  N=953 | GoSHARE (b)  N=88 | BREATHE  N=210 | Tayside RCT  N=62 | PAGES  N=163 | | GALA II  N=486 | SAGE  N=71 |
| Gender  (M vs F) | 1.07 (0.78-1.46) | 1.18 (0.76-1.82) | 12.9(2.10-79) | 1.04 (0.81-1.34) | 0.97 (0.31-2.71) | 1.99 (1.02-3.9); | | 1.16 (0.75-1.81) | 2.39 (0.74-7.76) |
|  | P=0.66 | P=0.44 | P=0.006* | P=0.74 | P=0.89 | P=0.04* | | P=0.494 | P=0.1465 |
| Age at 1^st^ LTRA | - | 1.01 (1.003-1.03) | 1.14(0.89-1.45) | - | - | - | | - | - |
|  | - | P=0.013* | P=0.29 | - | - | - | | - | - |
| Age at 1^st^ SABA | - | 1.01 (0.99-1.02) | 0.79 (0.56-1.09) | - | - | - | | - | - |
|  | - | P=0.18 | P=0.15 | - | - | - | | - | - |
| Exacerbation before 1^st^ LTRA | 1.4 (1.02-1.92) | 6.02 (3.91-9.26) | 8.91 (5.99-13.2) | - | - | - | | - | - |
|  | P<0.036* | P<0.0001* | P<0.0001* | - | - | - | | - | - |
| Age at recruitment | - | - | - | 0.86 (0.83-0.89) | 1.11 (0.96-1.29) | 0.93 (0.84-1.02) | | 0.03 (-0.01-0.07) | -0.07 ((-0.02) – (-0.18)) |
|  | - | - | - | P<0.0001* | P=0.14 | P=0.16 | | P=0.453 | P=0.476 |
| BMI | - | - | - | 0.95 (0.92-0.98) | 1.07 (0.94-1.22) | 0.97 (0.91-1.05) | | - | - |
|  | - | - | - | P=0.0035* | P=0.24 | P=0.54 | | - | - |
| Age of asthma onset | 1.06 (1.02-1.09) | - | - | - | - | - | | -0.07 [-0.10- (-0.03)] | -0.06 ((-0.25)-0.14) |
|  | P=0.0003* | - | - | - | - | - | | P=0.052* | P=0.5756 |
| PC1 | - | - | - | - | - | - | | 47.87 [-55.85-(-39.89)] | 19.24 ((-6.85)-45.32) |
|  | - | - | - | - | - | - | | P<0.0001* | P=0.148 |
| PC2 | - | - | - | - | - | - | | 5.28 (-2.14-12.71) | -0.60 ((-29.23)- 28.03) |
|  | - | - | - | - | - | - | | P=0.476 | P=0.967 |
