# Supplementary material for: LTA4H rs2660845 association with montelukast response in early and late-onset asthma
Source: PLoS One. 2021 Sep 22;16(9):e0257396. doi: 10.1371/journal.pone.0257396 (PMC8457475; doi:10.1371/journal.pone.0257396)
Supplement: S4 Table — 1Exacerbation within 6 months; 2Exacerbation within 12 months; OCS: Oral Corticosteroids; ER: Emergency Room visit; GoSHARE (a) is the late-onset population (>18 years-old); GoSHARE (b) is the early-onset population (< = 18 years-old). (DOCX) [file pone.0257396.s004.docx]

**S4 Table. Details of late-onset and early-onset in three non-montelukast user populations.**

|  | GoSHARE (a) | GoSHARE (b) | BREATHE | PAGES |
| --- | --- | --- | --- | --- |
| N | 953 | 88 | 94 | 356 |
| % male (n) | 44 | 21 | 57 | 56 |
| Mean age (SD) years | 40 (16) | 9 (5) | 10 (3.6) | 10 (3.5) |
| Study type | longitudinal | longitudinal | Cross-sectional | Cross-sectional |
| Exacerbation in 6-12 month | OCS, hospitalisation, ER^2^ | OCS, hospitalisation, ER^2^ | OCS, hospitalisation, school absence^1^ | OCS, hospitalisation, school absence^1^ |
| Exacerbation (%) | 19 | 12 | 36 | 61 |
| rs2660845 G variant frequency | 0.27 | 0.27 | 0.27 | 0.26 |

^1^Exacerbation within 6 months; ^2^Exacerbation within 12 months

OCS: Oral Corticosteroids

ER: Emergency Room visit

GoSHARE (a) is the late-onset population (>18 years-old)

GoSHARE (b) is the early-onset population (<= 18 years-old)
