# Supplementary material for: LTA4H rs2660845 association with montelukast response in early and late-onset asthma
Source: PLoS One. 2021 Sep 22;16(9):e0257396. doi: 10.1371/journal.pone.0257396 (PMC8457475; doi:10.1371/journal.pone.0257396)
Supplement: S5 Table — 1Exacerbation within 6 months; 2Exacerbation within 12 months; OCS: Oral Corticosteroids; ER: Emergency Room visit; Patients were diagnosed as having early-onset asthma; Montelukast prescription records were only available as adults. (DOCX) [file pone.0257396.s005.docx]

**S5 Table. Details of early-onset asthma adult montelukast user from the UKBiobank.**

|  | UKBiobank |
| --- | --- |
| N | 511 |
| % male (n) | 34 |
| Mean age (SD) years | 10 (4) |
| Study type | longitudinal |
| Exacerbation in 12 months | OCS, hospitalisation, ER |
| Exacerbation (%) | 21 |
| rs2660845 G variant frequency | 0.27 |

OCS: Oral Corticosteroids

ER: Emergency Room visit

Patients were diagnosed as having early-onset asthma.

Montelukast prescription records were only available as adults.
