# Supplementary material for: LTA4H rs2660845 association with montelukast response in early and late-onset asthma
Source: PLoS One. 2021 Sep 22;16(9):e0257396. doi: 10.1371/journal.pone.0257396 (PMC8457475; doi:10.1371/journal.pone.0257396)
Supplement: S6 Table — Patients were diagnosed as having early-onset asthma; Montelukast prescription records were only available as adults. (DOCX) [file pone.0257396.s006.docx]

**S6 Table.** **Association between rs2660845 and asthma exacerbation in early-onset UKBiobank individuals 12 months after taking montelukast prescription as adults.**

| Study | UKBiobank (n=511) |
| --- | --- |
| OR (95% CI) | 1.18 (0.86-1.61) |
| P-value | 0.283 |

Patients were diagnosed as having early-onset asthma.

Montelukast prescription records were only available as adults.
