# Supplementary material for: LTA4H rs2660845 association with montelukast response in early and late-onset asthma
Source: PLoS One. 2021 Sep 22;16(9):e0257396. doi: 10.1371/journal.pone.0257396 (PMC8457475; doi:10.1371/journal.pone.0257396)
Supplement: S7 Table — BIOSQTL: The Biobank-Based Integrative Omics Study Quantitative Trait Locus; eQTLGen: expression Quantitative Trait Loci Genetic; FDR: False Discovery Rate; ID: Gencode Identifier. (DOCX) [file pone.0257396.s007.docx]

**S7 Table. Cis-eQTL effect of rs2660845 on *LTA4H* expression in whole blood from adult cohorts.**

| Consortium | P-value | rsID | Chr | Position(hg19) | ID | Gene symbol | Z-score | Assessed | Other | Number of cohorts | Number of samples | FDR |
| --- | --- | --- | --- | --- | --- | --- | --- | --- | --- | --- | --- | --- |
| BIOSQTL | 6.27E-08 | rs2660845 | 12 | 96438553 | ENSG00000111144,ENSG00000257878 | LTA4H | -5.41 | G | A | 4 | 2116 | 0 |
| eQTLGen | 4.89E-07 | rs2660845 | 12 | 96438553 | ENSG00000111144 | LTA4H | -5.03 | G | A | 37 | 31683 | 0.0016 |

BIOSQTL: The Biobank-Based Integrative Omics Study Quantitative Trait Locus [1]

eQTLGen: expression Quantitative Trait Loci Genetic [2]

FDR: False Discovery Rate

ID: Gencode Identifier

**References**

1. Zhernakova DV, Deelen P, Vermaat M, Van Iterson M, Van Galen M, Arindrarto W, Van't Hof P, Mei H, Van Dijk F, Westra H-JJNg. Identification of context-dependent expression quantitative trait loci in whole blood. 2017: 49(1): 139.

2. Võsa U, Claringbould A, Westra H-J, Bonder MJ, Deelen P, Zeng B, Kirsten H, Saha A, Kreuzhuber R, Kasela SJb. Unraveling the polygenic architecture of complex traits using blood eQTL meta-analysis. 2018: 447367.
