# Supplementary material for: LTA4H rs2660845 association with montelukast response in early and late-onset asthma
Source: PLoS One. 2021 Sep 22;16(9):e0257396. doi: 10.1371/journal.pone.0257396 (PMC8457475; doi:10.1371/journal.pone.0257396)
Supplement: S8 Table — 1 Patients were diagnosed as having early-onset asthma but with montelukast prescription records only available as adults. 2 GoSHARE individuals with exacerbation events in a year before first prescription of montelukast. 3 Individuals from BREATHE and PAGES cohorts not under montelukast treatment. GoSHARE(a): individuals with age at first salbutamol, age at first inhaled corticosteroid and age at first montelukast prescription, all three over 18 years old. GoSHARE(b): individuals with age at first salbutamol, age at first inhaled corticosteroid and age at first montelukast prescription, all three under or at 18 years old. (DOCX) [file pone.0257396.s008.docx]

**S8 Table. Genotype counts for rs2660845 SNP by exacerbation status**

| Status | Non-exacerbators | | | Exacerbators | | |
| --- | --- | --- | --- | --- | --- | --- |
| Cohorts \| rs2660845 genotypes | AA | AG | GG | AA | AG | GG |
| UKB | 742 | 518 | 104 | 110 | 69 | 18 |
| UKB early-onset asthma group, adult montelukast user ^1^ | 242 | 170 | 30 | 34 | 29 | 6 |
| GoSHARE(a) | 458 | 331 | 50 | 64 | 41 | 9 |
| GoSHARE(a) individuals 12 months before being on montelukast ^2^ | 458 | 331 | 50 | 64 | 41 | 9 |
| GoSHARE(b) | 57 | 24 | 1 | 1 | 3 | 2 |
| GoSHARE(b) individuals 12 months before being on montelukast ^2^ | 57 | 25 | 0 | 2 | 4 | 0 |
| BREATHE | 122 | 64 | 7 | 5 | 9 | 3 |
| BREATHE non-montelukast users ^3^ | 3 | 14 | 20 | 5 | 23 | 29 |
| Tayside RCT | 20 | 14 | 0 | 15 | 13 | 0 |
| PAGES | 26 | 21 | 7 | 64 | 39 | 6 |
| PAGES non-montelukast users ^3^ | 127 | 86 | 14 | 68 | 52 | 9 |
| GALA II | 41 | 58 | 36 | 112 | 164 | 75 |
| SAGE | 4 | 13 | 2 | 26 | 24 | 2 |

^1^ Patients were diagnosed as having early-onset asthma but with montelukast prescription records only available as adults.

^2^ GoSHARE individuals with exacerbation events in a year before first prescription of montelukast.

^3^ Individuals from BREATHE and PAGES cohorts ^not^ under montelukast treatment

GoSHARE(a): individuals with age at first salbutamol, age at first inhaled corticosteroid and age at first montelukast prescription, all three over 18 years old.

GoSHARE(b): individuals with age at first salbutamol, age at first inhaled corticosteroid and age at first montelukast prescription, all three under or at 18 years old.
