# Supplementary figures and images for: LTA4H rs2660845 association with montelukast response in early and late-onset asthma
Source: PLoS One. 2021 Sep 22;16(9):e0257396. doi: 10.1371/journal.pone.0257396 (PMC8457475; doi:10.1371/journal.pone.0257396)

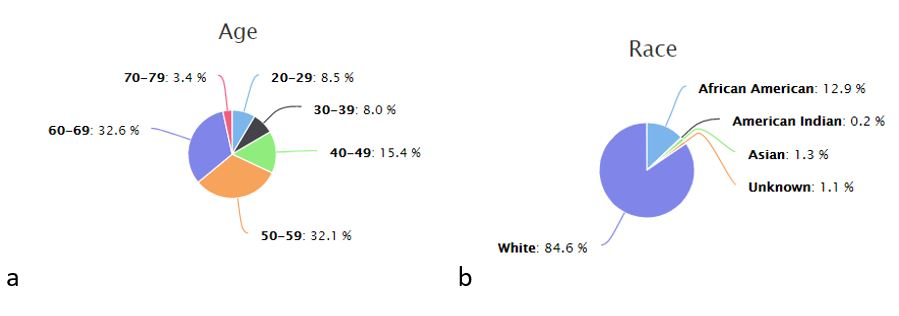

Supplement: S1 Fig — Age (a) and ethnicity (b) distributions in the GTEx portal (V8). (TIF) [file pone.0257396.s009.tif]

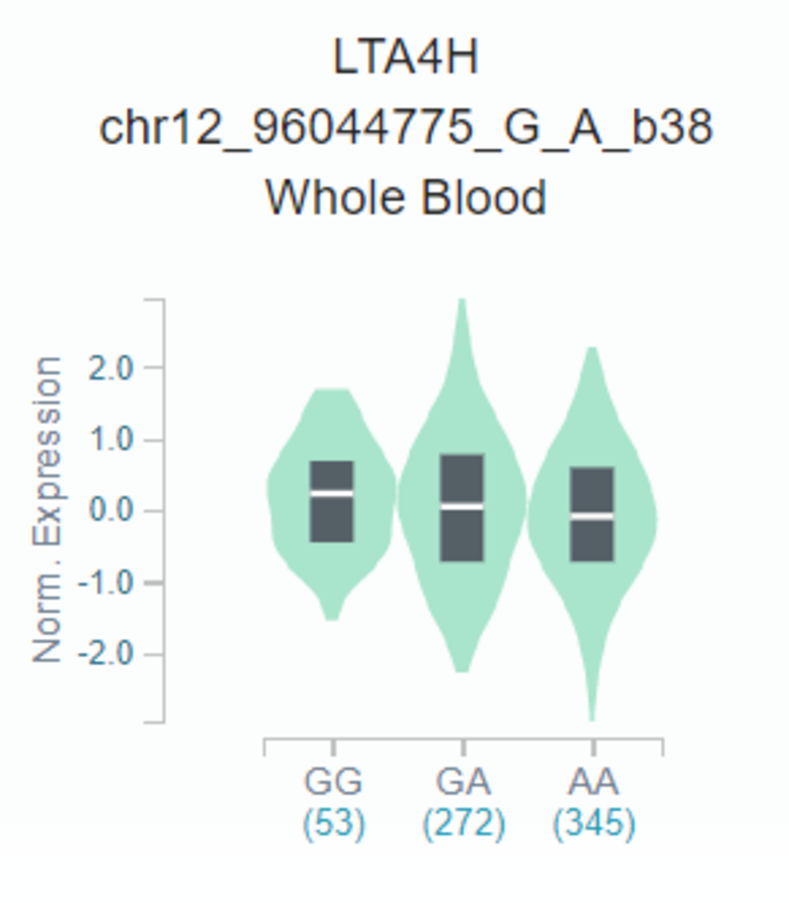

Supplement: S2 Fig — (GTEx v8). (TIF) [file pone.0257396.s010.tif]
